# Unveiling the Connection between Microbiota and Depressive Disorder through Machine Learning

**DOI:** 10.3390/ijms242216459

**Published:** 2023-11-17

**Authors:** Irina Y. Angelova, Alexey S. Kovtun, Olga V. Averina, Tatiana A. Koshenko, Valery N. Danilenko

**Affiliations:** Vavilov Institute of General Genetics, Russian Academy of Sciences (RAS), 119333 Moscow, Russia; kovtunas25@gmail.com (A.S.K.); olgavr06@mail.ru (O.V.A.); valerid@vigg.ru (V.N.D.)

**Keywords:** depression, gut microbiota, gut–brain axis, whole metagenome, neuroactive metabolites, signature, dysbiosis, biomarkers, machine learning

## Abstract

In the last few years, investigation of the gut–brain axis and the connection between the gut microbiota and the human nervous system and mental health has become one of the most popular topics. Correlations between the taxonomic and functional changes in gut microbiota and major depressive disorder have been shown in several studies. Machine learning provides a promising approach to analyze large-scale metagenomic data and identify biomarkers associated with depression. In this work, machine learning algorithms, such as random forest, elastic net, and You Only Look Once (YOLO), were utilized to detect significant features in microbiome samples and classify individuals based on their disorder status. The analysis was conducted on metagenomic data obtained during the study of gut microbiota of healthy people and patients with major depressive disorder. The YOLO method showed the greatest effectiveness in the analysis of the metagenomic samples and confirmed the experimental results on the critical importance of a reduction in the amount of *Faecalibacterium prausnitzii* for the manifestation of depression. These findings could contribute to a better understanding of the role of the gut microbiota in major depressive disorder and potentially lead the way for novel diagnostic and therapeutic strategies.

## 1. Introduction

Depression, as a widespread and destructive mental disorder, remains one of the most important health challenges of our time (WHO: 5% of adults suffer from depression). Scientific research is normally focused on the biochemical and neural aspects of depression, without taking into account the potential impact of the gut microbiota. The study of intestinal microbiota metabolites affecting mental health is becoming increasingly important in light of the possible relationship between the composition of microorganisms in the intestine and the functioning of the brain [1,2]. New research suggests that the state of the microbiota can affect neurotransmitters, inflammation and the immune system, which, in turn, has an impact on a person’s mental health [3]. Such discoveries can shift the focus of the study of depression [4] from mainly neurochemical and neurological aspects to the relationship between the intestinal microbiota and mental health [5]. In this context, understanding the role of the microbiota opens up new prospects for the development of more effective methods for the diagnosis and treatment of depression and other mental disorders [2,3,5].

The gut microbiota consists of an enormous number of microorganisms living in the human digestive tract and is a complex and dynamic ecosystem. Bacteria in the gut microbiota are not only involved in digestion and metabolic processes but also interact with the brain through a bidirectional signaling network known as the ‘gut-brain axis’ [6,7,8]. In this context, the brain exists in an interacting symbiosis with gut microbiota, and disorders in this complex system can have a significant impact on human mental health [9,10]. The identification and analysis of these relationships are complex tasks, requiring the processing and interpretation of huge amounts of data obtained using high-throughput sequencing methods.

The concept of a metagenome signature (the gene of a bacterium and its taxonomy) was previously introduced and has become generally accepted [10,11]. This made it possible to characterize the complex structure of the gut microbiota in its normal state [10]. At the next stage, the concept of functional architecture of the disease was introduced [12]. Generally, the scientific and technological base was prepared for the application of machine learning methods [13]. In our previous work [14], taxonomic and functional changes in the gut microbiota that correlate with depression in patients from Moscow and the Moscow region were identified using data obtained from whole metagenome sequencing. Metagenomic signatures of the gut microbiota were determined, describing the bacterial origin and changes in the abundance of the genes involved in the synthesis and metabolism of neuroactive compounds and biomarkers of depression. The results obtained in this study confirm the importance of the role of the gut microbiota in the formation of the neurometabolic profile and its connection with mental health. The novel knowledge on the gut microbiota and its metabolic products in the context of depression opens up prospects for the development of new methods for the diagnostics and treatment of this disorder based on the role of the microbial factor. Progress in this regard has been achieved thanks to the development of new algorithms in the analysis of metagenomes of the studied cohorts of patients.

Today, machine learning methods can bring scientific research up to a new level, providing powerful algorithms and tools for effective analysis, classification and prediction of the links between gut microbiota and depression [14,15]. There are a number of algorithms that are used to solve the problem of the association between the disease and the gut microbiota. They include random forest, logistic regression, neural networks, support vector machine, etc. [16].

Logistic regression is a generalized linear method that finds its application in binary classification problems. Recent studies, such as the work by Wu and co-authors (2022), successfully applied logistic regression to predict breast cancer and nonmalignant breast disease based on microbiome data [17]. The random forest algorithm was demonstrated to have high efficiency in microbial community studies. For example, it was used for the analysis of the immune response of intestinal microbiota [18]. The support vector machine has previously shown possibilities for use in classification tasks of metagenomic data [19]. However, some studies do not recommend using it with the microbiome data [16]. For this purpose, methods like lasso or elastic network are applied more often [16]. In recent years, various methods of deep learning have been used in metagenomic research, such as neural networks for classification and the construction of models for prediction [20,21,22,23,24,25]. For example, the proposed neural network was used as a classifying algorithm in a program for the prediction of the presence of the disease or its absence [23]. The tool requires large datasets, and it is applied on both 16S rRNA data and whole metagenome sequencing data. Another class of deep learning algorithms could be used for image classification tasks [22]. One of them is called the You Only Look Once (YOLO) algorithm [21]. It is a state-of-the-art object detection system that aims to predict the class of an object and the bounding box that defines the object location on the input image. This method has proven itself in detection and classification tasks [25,26].

The choice of the classification algorithm for the metagenomic study depends on the characteristics of the data, available resources and research objectives. Normally, the best results can be achieved by combining several methods. There are a number of problems that researchers face [26]. The first one is the quality of the data. Metagenomic data are often noisy and contain a lot of missing information, which can make it difficult to obtain accurate results [27]. The second problem is the complexity of the data: metagenomic data contain a large number of variables (either taxa or genes), which can make it difficult to identify significant patterns [27]. The next problem is the class imbalance; in metagenomic studies, the number of samples taken from healthy people often far exceeds the number of samples taken from patients with the disease. This can lead to class imbalance, which can make it difficult to prepare machine learning models that accurately predict disease status [27]. The identification of a causal relationship is an issue to be resolved in the future. Machine learning models are good at identifying correlations between variables, but they cannot determine causality. This means that even if the model reveals a strong link between particular traits of microbiota and the disease, it does not necessarily mean that the changes in microbiota were the cause of the disease [27]. Also, the interpretation of the results of application of the machine learning algorithms to the association tasks and understanding of how the predictions have been made can be difficult. This can become a problem when trying to determine the biological mechanisms underlying the links between microbiota and diseases [28]. The next problem is the dependence on training data, which is connected to the possibility of the occurrence of errors when applying the machine learning algorithms to the metagenomic data. For example, supervised learning models depend on the quantity and quality of the training data. This can be a problem if there are limited data or if the data quality is low [15]. In general, although machine learning can be a powerful tool for analyzing metagenomic data, it is important to be aware of these problems and carefully consider the limitations of the approach.

In this paper, the problem of classifying the depressive state of patients by the functional characteristic of gut microbiota (metagenomic signature) is solved using various machine learning methods: random forest, elastic network and YOLO. For this purpose, data from the sequencing of the whole metagenome are used.

## 2. Results

### 2.1. Selection of Features

Before application of the random forest and the elastic net algorithms for model training, the selection of features was conducted using the random forest based on the Gini criterion, as described in Section 4. For this purpose, metagenomic signatures identified in a previous study [14] were utilized (Supplementary Table S1). The resulting most significant signature pairs are presented in Table 1. It is noteworthy that among the listed signature pairs, a bacterium of the species *Faecalibacterium prausnitzii* prevails. A similar result was observed in our previous article [14]. The set of enzymes obtained during the selection of the traits also repeats the previously observed results. These enzymes are involved in the synthesis of spermidine, glutamate and asparagine. Five additional signature pairs were also selected as significant features by the random forest algorithm. They include enzymes involved in the formation of acetic and butyric short-chain fatty acids, inositol, diacylglycerol and arginine.

### 2.2. Machine Learning with Random Forest and Elastic Net

The identified significant features were used to train the models to distinguish between the microbiota of the healthy subjects and patients with depression. In the initial study, the cohort of the healthy volunteers was named ‘HC’ and the group of patients with depression was called ‘PwD’. Thus, this nomenclature is used in the current work.

**Figure 1:**
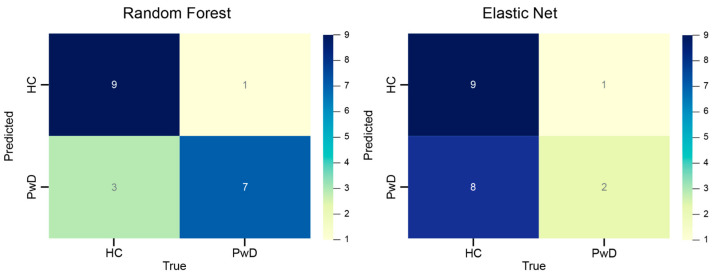
Confusion matrix: (**left**) random forest, (**right**) Elastic net. The ‘HC’ stands for ‘Healthy Controls’ and ‘PwD’ is ‘Patients with Depression’.

Classical machine learning algorithms were applied: random forest and elastic network. For this purpose, the initial cohort was split into two subgroups, 30 percent of the samples for the testing dataset and 70 percent for the training dataset. The characteristics of the resulting training models are presented in Table 2, and the results of the models’ evaluation on the test dataset are shown in Figure 1. The model obtained by the random forest algorithm showed good overall results: the percentage of false-positive predictions was 10%, and false negative-predictions were around 30%. This accuracy is consistent with the results obtained in other metagenomic studies using random forest [29]. Application of the elastic net algorithm for this task gave worse results. While the percentage of false-positive predictions was at 10%, which is similar to random forest, the rate of false negatives, on the other hand, was significantly higher at 80%.

### 2.3. Classification of Metagenomic Samples with YOLO

**Figure 2:**
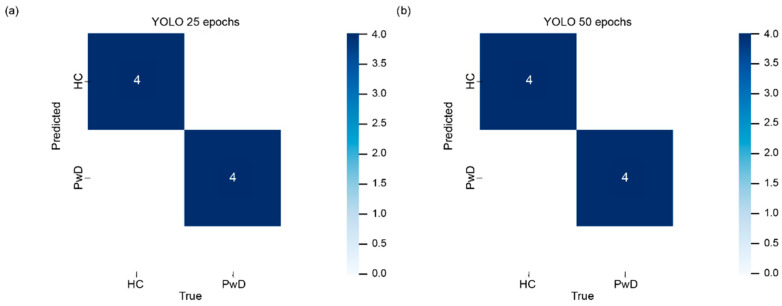
The Confusion matrix compiled for the test dataset displays the number of false positive and false negative results when training YOLO at 25 epochs (**a**) and 50 epochs (**b**). The ‘HC’ stands for ‘Healthy Controls’ and ‘PwD’ is ‘Patients with Depression’.

Neural networks can be considered as another approach for solving the problem of metagenomic samples’ classification. Since metagenomic signatures can be conveniently represented in the form of heat maps, the image classification approach using YOLO was chosen. Before training the YOLO model, a set of artificial data was obtained: 900 for the HC group and 900 for the PwD. The total size of the final dataset accounted for 1874 samples, 938 in the HC group and 936 in the PwD group. More information about the dataset can be found in Supplementary Table S1. Since each sample is a text data table, and YOLO is an image processing algorithm, all samples were transformed into heat maps that display the abundance value using color gradient. After that, the training of models with YOLO was conducted in 25 epochs and 50 epochs independently. The resulting characteristics of the obtained models are presented in Table 3. The metrics show very high accuracy, precision (positive predictive value, PPV) and recall (sensitivity) rates at 100% for both 25 and 50 epochs. The accuracy of the models fluctuated greatly in the early stages of training and set at a final value approximately after the 25th epoch (Supplementary Figure S1 and Supplementary Table S2a). During the subsequent epochs, the fluctuations in the accuracy rate were slight, and they mostly remained at the highest level, which indicates no necessity for a further increase in training rounds (Supplementary Figure S2 and Supplementary Table S2b). The false-positive and false-negative rates were at 0 percent (Figure 2). The accuracy of the resulting models for both 25 and 50 epochs was also examined in the dedicated test dataset. The average accuracy of the YOLO algorithm in the testing samples was 87% for 25 epochs and 94% for 50 epochs (Table 4).

### 2.4. Validation of YOLO Learning Outcomes

The YOLO classification method is based on the description of the general characteristics of the image: the location of its outlines and colors. During model training, the rows and columns in the signature tables were sorted alphabetically, which, in theory, can lead to a certain bias. To check whether there is a possible dependence on the order of the parameters in the initial matrices, a validation procedure was performed, which included 10 rounds of random permutations and retraining of the models. The results of each round of testing can be seen in Supplementary Figures S3–S12. It was confirmed that the proposed method demonstrates high efficiency, regardless of the location of rows and columns in the source data with the smallest accuracy value of 88% (Supplementary Table S3). This shows the potential of the YOLO algorithm for the application in metagenomic studies, particularly for the classification of metagenomic signatures associated with depressive disorder, and it emphasizes the prospects of this method for such analytical tasks.

## 3. Discussion

The use of modern sequencing technologies for metagenomic studies and machine learning methods opens up new prospects for identifying complex relationships between gut microbiota and mental health, which may be important for developing innovative approaches for the diagnostics and treatment of diseases. It is interesting to note that the changes in the composition of gut microbiota in patients with major depressive disorder are associated with disturbances in the neurometabolic profile [28]. These connections, observed via metagenomic signatures [10,11,14], reveal the correlations between the conditions of patients with depression and the altered abundance of bacterial genes involved in the production of short-chain fatty acids and neurotransmitters, including serotonin, GABA and glutamate and other biomarker neurometabolites [1,11]. Such an effect of gut microbiota on the overall level of these metabolites in the body may play an important role in the pathophysiology of depression and may represent promising goals for future research and treatment.

In this research, metagenomic data were analyzed using various machine learning algorithms to identify associations between gut microbiota and depression. During the course of the study, a set of significant functional features, metagenomic signatures at the taxonomic level of species, characterizing the key differences between the gut microbiota of patients with a depressive state from healthy people was determined using random forest machine learning algorithm methods. Similar characteristics were described in earlier studies [10,14]; however, they generally relied on classical methods of statistical analysis. Arguably one of the most remarkable traits of the described significant features defining the depressed condition is the presence of one particular species, *Faecalibacterium prausnitzii*, in signature pairs with a large number of genes [30,31,32].

*F. prausnitzii* is one of the most common microbial species in the colon of healthy adults and constitutes over 5–15% of the overall total bacterial population [33]. *Faecalibacterium* is prevalent in human populations across the world—it was detected in 85% of gut samples [34]. In various studies, it was shown that patients with major depressive disorder had decreased abundance of the bacteria of the *Faecalibacterium* genus in their gut microbiota compared to control groups [10,14,15,31]. *Faecalibacterium* is considered one of the key producers of short-chain fatty acids, such as butyrate [34]. Short-chain fatty acids are important for intestinal health, because they serve as the main source of energy for the cells of the intestinal wall and contribute to healthy functioning. In addition, *Faecalibacterium* can have an anti-inflammatory effect by reducing the level of inflammatory cytokines in the intestine [33,34]. The link between the reduced *Faecalibacterium* levels and depression may be associated with the violation of the balance of gut microbiota, which may affect neuroinflammation and neuromodulation through complex signaling mechanisms [34,35]. The changes in the composition of gut microbiota, including the decrease in levels of *Faecalibacterium*, can lead to an imbalance of neurotransmitters and inflammatory mechanisms, which, in turn, will affect mood and mental state in patients with depression [34,36,37]. However, one should mention that the mechanisms of interaction between the bacteria of the *Faecalibacterium* genus and depression remain poorly understood and require additional research to establish a causal relationship. Still, the results of this study provide additional evidence about the important role of the gut microbiota in the pathophysiology of the depressive condition and may contribute to the development of understanding of this disease and, more importantly, its relations with gut bacteria.

Random forest is one of the most widely used methods in machine learning and allows for the creation of models with relatively high accuracy rates. One of the reasons for that is the ability of the random forest algorithm to take a large number of features into account. It is also resistant to the presence of noise and emissions in the training dataset, which makes it a common choice for modern metagenomic studies. In this paper, random forest was applied for the construction of models that could potentially define the depressed condition through the traits of the metagenomic signatures of gut microbiota in the patient. Similar research was conducted to describe the changes in functional and microbial composition of the gut microbiota in patients with colorectal cancer using random forest [38]. The constructed models showed great results during validation and were proposed as novel predictors for the diagnostics of the disease. The application of random forest allowed us to obtain models that provide high prediction accuracy and a small percentage of false-positive predictions, which is consistent with the results shown in other works. The poor results of the elastic network can be explained by the features of the metagenomic data (such as a large number of outliers). Also, the quality of classification could be affected by an insufficient amount of real data, which led to such low accuracy rates. Successful applications of this method are described in the literature but, usually, the elastic network shows results worse than those obtained by random forest [27].

A distinctive feature of our work compared to previous studies is the usage of the YOLO algorithm for the classification of gut microbiota samples by metagenomic signatures as functional characteristics. The resulting models showed better accuracy and false-positive rates than the ones obtained by the random forest algorithm, as it allowed us to consider the patterns in the gut microbiota. Of course, the reason for such high rates of the models generated by YOLO is also related to the fact that the number of real sequenced samples in the dataset was not large, and the generation of synthetic samples was required for training. Validation with the help of additional rounds of analysis after random permutations in the source data allowed us to confirm the effectiveness of the obtained models. Even after permutations, the average accuracy rate of the YOLO models remained higher than the ones generated by random forest. Of course, to improve the performance of YOLO and the quality of the predictions obtained with its models, it is necessary to expand the sample of real data and then retrain the model, which will be one of the key aims of our future research.

Another trait of the applied YOLO algorithm is the fact that the current version of this method provides only general classification for the samples based on all found traits; in our case, this was metagenomic signatures, which are associated with the depressive disorder. In the future, it will be possible to determine specific signature pairs, on the basis of which the most accurate forecast can be created using this algorithm.

## 4. Materials and Methods

### 4.1. Studied Cohort and Data Structure

In this work, data from metagenomic signatures from the previous article (BioProject ID: PRJNA762199) were used, where changes in gut microbiota correlating with the depressive state of patients from Moscow and the Moscow region were determined using classical statistical analysis with Wilcoxon rank test and the FDR correction with the Benjamini–Hochberg method [14]. The cohort consists of 74 people aged 18 to 54 years from Moscow and the Moscow region, including 38 healthy volunteers and 36 patients with a diagnosed depressive disorder. In this paper, the obtained metagenomic signatures are used for further application of the machine learning classification methods. Metagenomic signatures are the tables showing the genes found in each sample, their bacterial origin at the species taxonomic level determined using the Kraken2 tool version 2.1.2 [39] and abundance (Supplementary Table S1).

### 4.2. Machine Learning Methods for Metagenomic Classification

In this paper, various machine learning algorithms are used to describe changes in the metagenomic signature that characterize the state of the gut microbiota during depression, as well as to construct a classifier of the patients’ mental state based on their metagenomic signature. Each algorithm was applied to train models for classifying samples into groups of ‘patients with depression’ (PwD) and ‘healthy controls’ (HC), as well as to determine the most informative signatures associated with depression.

#### 4.2.1. Classical Machine Learning Methods

The selection of features for classification was carried out with the random forest method. It uses the Gini criterion, or average uncertainty reduction, to assess the importance of features in modeling, based on how much their exclusion affects the accuracy of the model, and, thus, determine which features are most important. For this purpose, the function RandomForestClassifier() from the sklearn library was used [40]. This method helped identify and extract the most significant features from the dataset, which were then utilized as attributes for training various machine learning algorithms. Then, the obtained significant features were used as attributes for training of the machine learning algorithms. The fundamental classical methods of machine learning, the random forest and the elastic net method, were utilized for the classification analysis. The RandomForestClassifier() and LogisticRegression() with ‘elasticnet’ setting functions were used to train the models.

The main emphasis was placed on the identification of informative metagenomic signatures related to the pathological condition of a depressive nature, as well as on the formation of classification models. Evaluation of the effectiveness of the generated models was carried out using the metrics toolkit built into the Python scikit-learn software package version 1.1.1 [40].

#### 4.2.2. You Only Look Once Method

**Figure 3:**
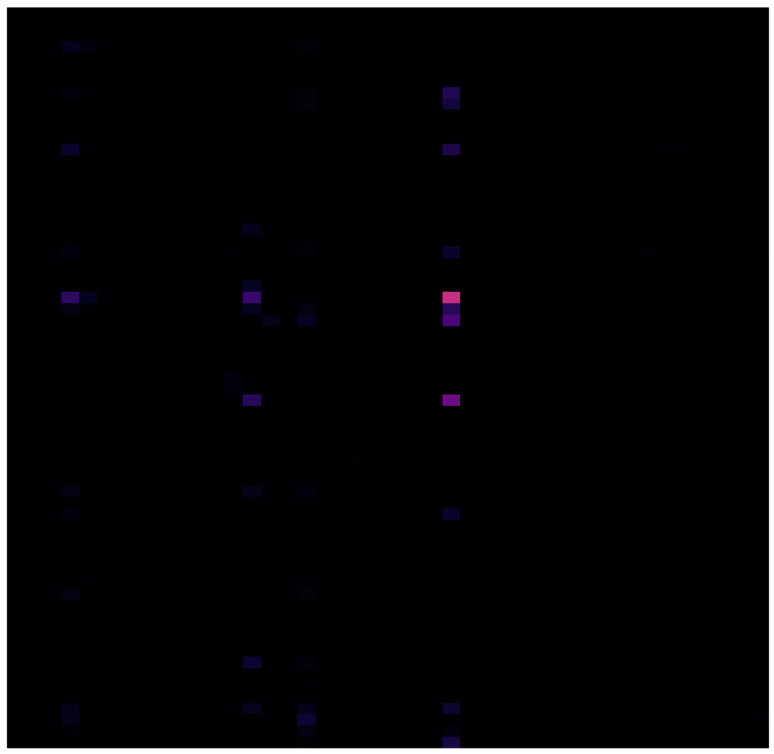
Example of graphical representation of metagenomic signature with gradients. Bacteria are in the rows, and genes are in the columns.

Ultralytics YOLOv8 provides models for object detection, instance segmentation and image classification tasks. For image classification, YOLOv8 Classify models are used, which classify an entire image into one of a set of predefined classes. In order to ensure the effectiveness of the training of the model and the subsequent evaluation of its performance, a test subset consisting of 4 healthy volunteers and 4 patients with depression was isolated from the initial dataset. Then, a synthetic dataset was constructed based on the remaining data using the toolkit Mostly.ai version 109 (https://mostly.ai/, accessed on 21 July 2023) [41]. The tool is used through the website interface. The synthetic samples were saved in the format of text tables, which included signature pairs (enzyme; bacterial species) and their abundances. The samples were then transformed into images representing heat maps, in which the rows correspond to the enzymes, the columns to bacterial species and the color gradient to the abundance of the signature pairs. The black color was chosen as the base one (zero abundance) (Figure 3).

Next, the resulting set of the real and simulated data was divided into two groups: training and validation. This was necessary for training the YOLO model. Next, the ultralytics library with the implementation of the YOLO v8 algorithm [42,43] was used to train the algorithm (implementation of YOLO in code: https://colab.research.google.com/drive/1ZT-6oRWYmaE1HLbvYygom_gn7eFg5P7z?usp=sharing, accessed on 21 July 2023). Accuracy, precision (positive predictive value, PPV), recall (sensitivity) and f1-score were applied as training characteristics [22]. They were calculated using the following formulas:

$$accuracy=\frac{\left(TP+TN\right)}{\left(P+N\right)}$$

$$precision=\frac{TP}{\left(TP+FP\right)}$$

$$recall=\frac{TP}{\left(TP+FN\right)}$$

$$f1score=\frac{2\cdot precision\cdot recall}{precision+recall}$$

The training parameters were selected in accordance with the recommendations of the developers of the YOLO algorithm [43], as presented in Table 5.

### 4.3. Validation of the Method

In order to confirm the independence and reliability of the proposed method, additional validation experiments were conducted. The columns and rows were mixed in the initial samples; then, the model was retrained, and its prediction accuracy was tested. This cycle of training and validation was repeated in ten independent iterations with training on 25 epochs, which contributed to the confirmation and approval of the quality and effectiveness of the proposed methodology.

## 5. Conclusions

To date, methods for diagnosing depressive disorder in patients have mainly relied on the usage of questionnaires, observation of their general physical state by the therapist, analysis of biochemistry, MRI, etc. [43,44,45,46]. The described functional changes in gut microbiota associated with depression make the metagenomic signatures a novel method for the diagnostics and monitoring of the disease, where the signature pairs can be utilized as defining biomarkers. The modern methods of machine learning allowed us to create the prediction models, which can be applied to a sequenced metagenomic sample and predict the mental condition of an individual based on the taxonomic and functional features of the gut microbiota. These models can be utilized for the in silico diagnostics of major depressive disorder in addition to existing methods. Another possible application of such algorithms of rapid diagnostics is the development of new methods of treatment for depression by the correction of gut microbiota with the use of psychobiotics [47,48,49,50]. Further, machine learning methods should also be utilized for the integrated analysis of metagenomic signatures and biochemical, immunological and neurological biomarkers, i.e., functional architecture of the disease [12].

## Figures and Tables

**Table 1:** Selected features for model training. The table shows the metagenomic signature pairs obtained as a result of the selection of significant features by the random forest method. Signature pairs, in which a statistically significant change was shown in a previous study [14], are highlighted with bold font.

Species	Enzyme
*Bacteroides caccae*	Phosphotransacetylase
*Coprococcus comes*	Argininosuccinate lyase
*Eggerthella lenta*	Butyryl-CoA-dehydrogenase
*Escherichia coli*	Diacylglycerol kinase
*Escherichia coli*	Myo-inositol-1(or 4)-monophosphatase
***Faecalibacterium prausnitzii***	**Asparagine synthetase (asnA)**
***Faecalibacterium prausnitzii***	**Glutamate synthase gltB**
***Faecalibacterium prausnitzii***	**Glutamate synthase gltD**
***Faecalibacterium prausnitzii***	**Spermidine synthase**

**Table 2:** Characteristics of the models trained by random forest and elastic net.

Model	Accuracy	Precision (Positive Predictive Value, PPV)	Recall (Sensitivity)	F1-Score
Random forest	0.80	0.75	0.90	0.82
Elastic net	0.55	0.53	0.90	0.67

**Table 3:** Characteristics of models trained by YOLO on 25 and 50 epochs.

YOLO Model	Accuracy	Precision (Positive Predictive Value, PPV)	Recall (Sensitivity)	F1-Score
25 epochs	1.00	1.00	1.00	1.00
50 epochs	1.00	1.00	1.00	1.00

**Table 4:** Prediction using the YOLO (25 epochs and 50 epochs) class model for samples from the test dataset. The ‘HC’ stands for ‘Healthy Controls’ and ‘PwD’ is ‘Patients with Depression’.

Sample ID	Accuracy (25 Epochs)	Accuracy (50 Epochs)
HC_1	0.90	1.00
HC_2	0.71	0.99
HC_39	1.00	1.00
HC_40	1.00	1.00
PwD_2	0.81	0.79
PwD_3	0.53	1.00
PwD_36	1.00	0.78
PwD_37	0.99	1.00

**Table 5:** Parameters for training at 50 epochs.

Parameter	Value
Image Size	1280 × 1280
Optimizer	AdamW (lr = 0.0007, momentum = 0.9)
Batch Size	4
Number of epochs	50

## Data Availability

The metagenomic samples used in this research are available on the NCBI SRA database under BioProject ID PRJNA762199. YOLO model used in this research is available under: https://colab.research.google.com/drive/1ZT-6oRWYmaE1HLbvYygom_gn7eFg5P7z?usp=sharing (accessed on 21 July 2023).

## Supplementary Materials

The following supporting information can be downloaded at: https://www.mdpi.com/article/10.3390/ijms242216459/s1.
