# Supplementary material for: Unveiling the Connection between Microbiota and Depressive Disorder through Machine Learning
Source: Int J Mol Sci. 2023 Nov 17;24(22):16459. doi: 10.3390/ijms242216459 (PMC10671666; doi:10.3390/ijms242216459)
Supplement: Supplementary file 1 [file ijms-24-16459-s001.zip › Supplementary Figures.pdf]

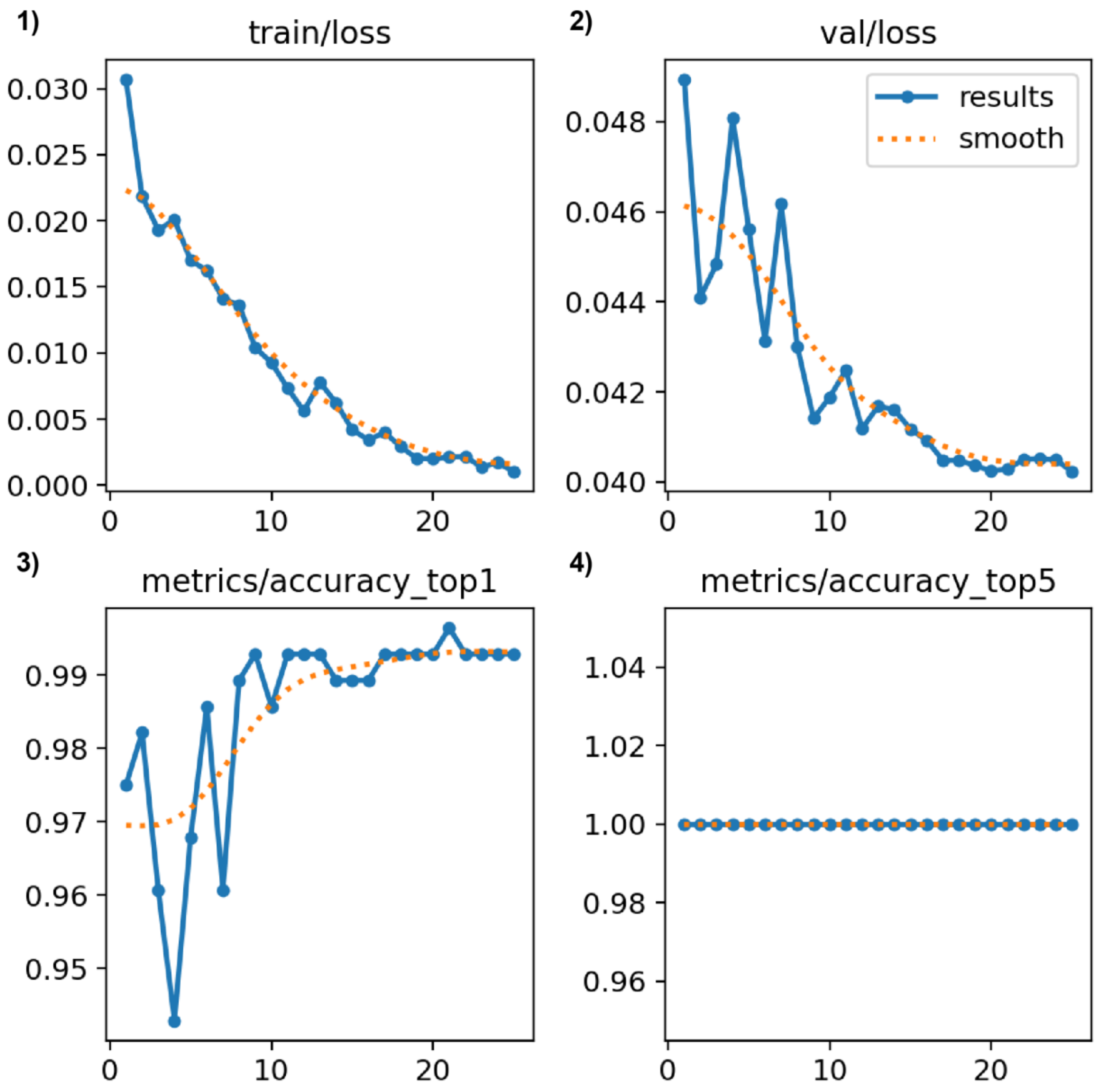

Figure S1. YOLO training on 25 epochs. 1) - 2) Loss curves for the model (the loss variation curves for the models). 3) - 4) Quality change during the learning process.

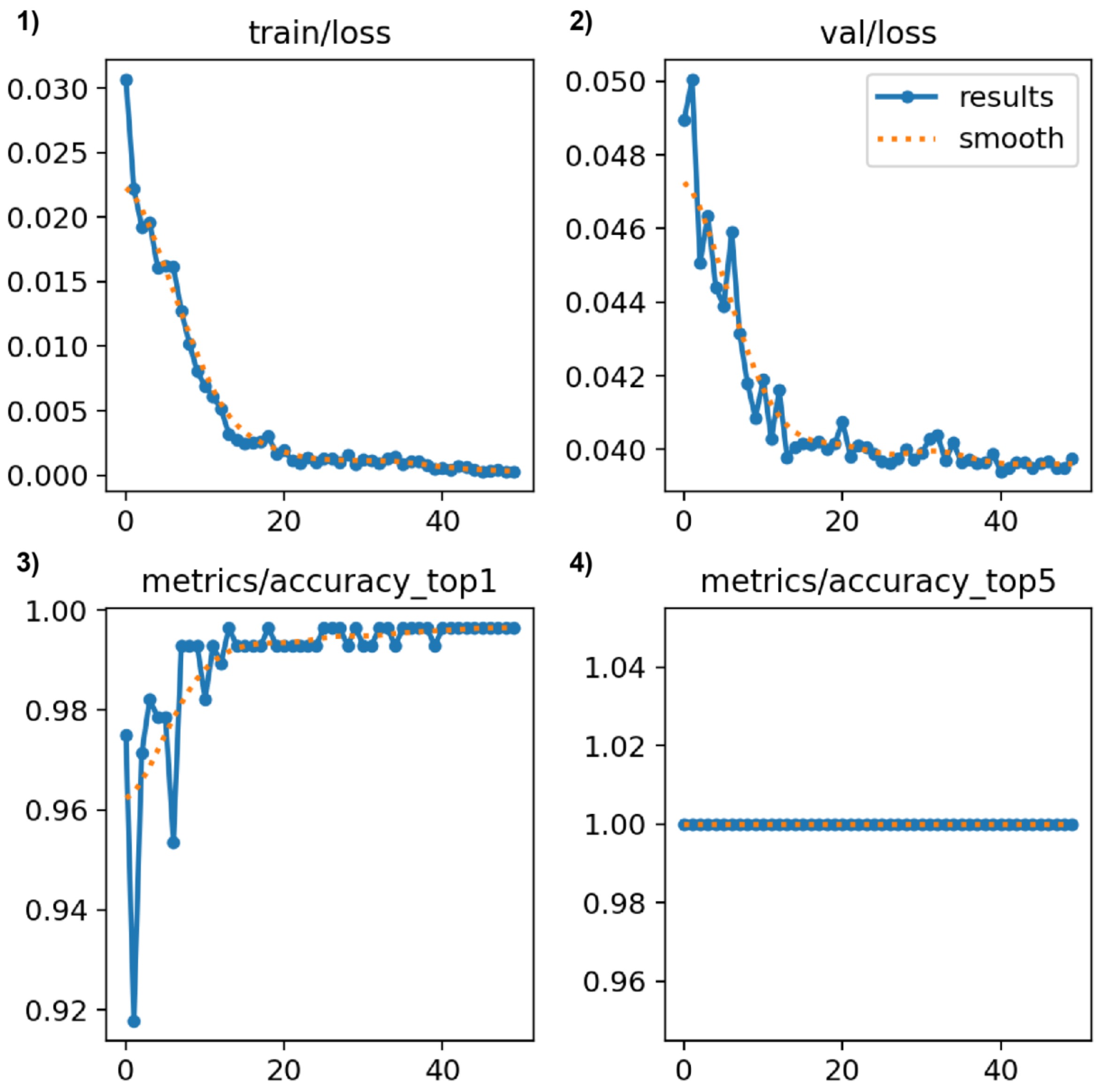

Figure S2. YOLO training on 50 epochs. 1) - 2) Loss curves for the model (the loss variation curves for the models). 3) - 4) Quality change in the learning process.

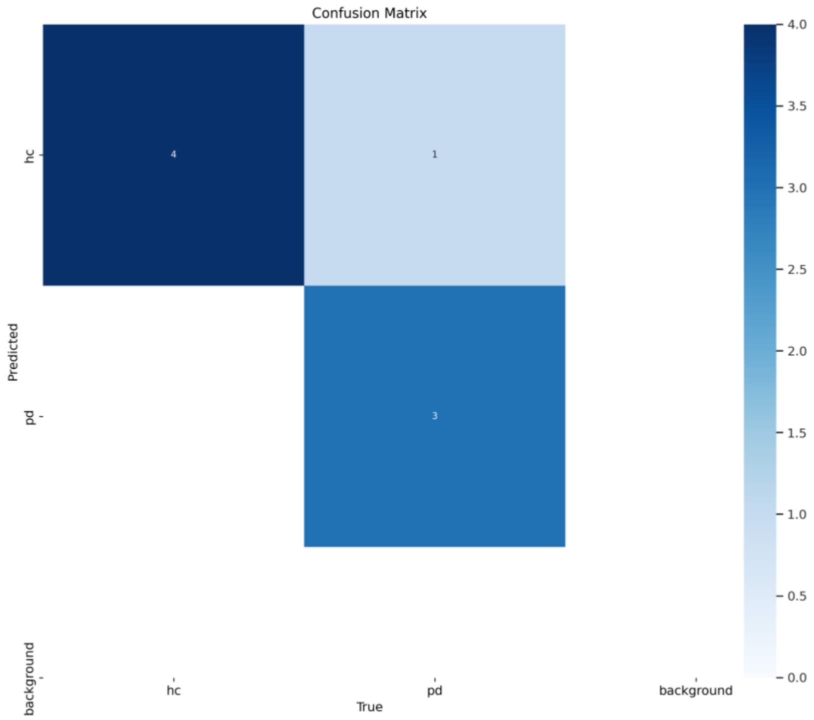

Figure S3. Confusion matrix of YOLO for test dataset for the 1st round of permutations.

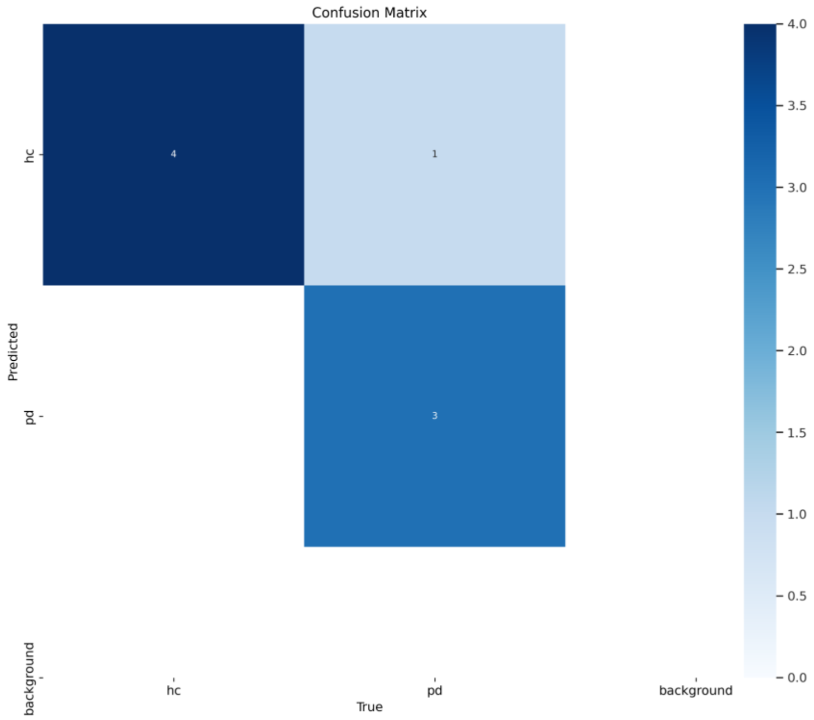

Figure S4. Confusion matrix of YOLO for test dataset for the 2nd round of permutations.

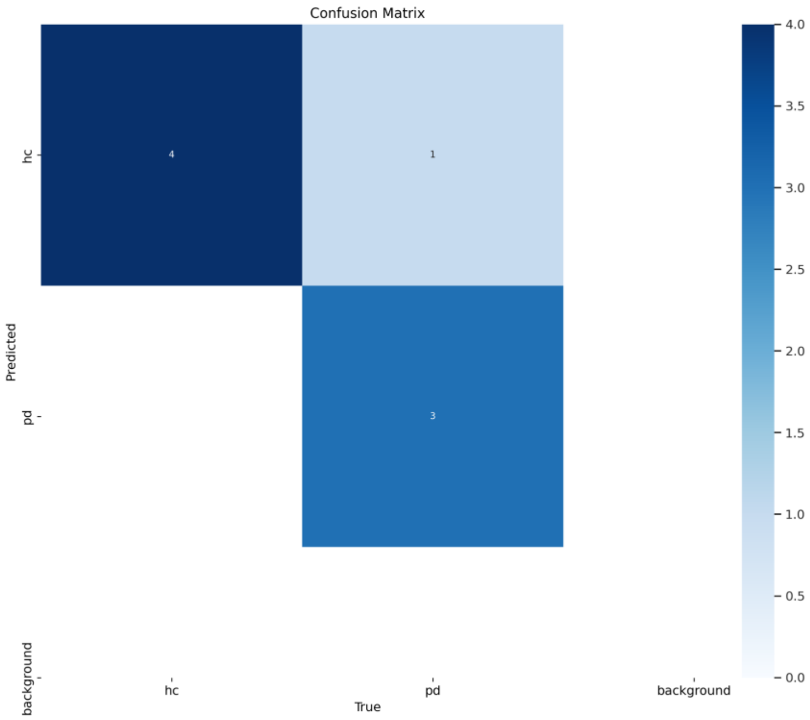

Figure S5. Confusion matrix of YOLO for test dataset for the 3rd round of permutations.

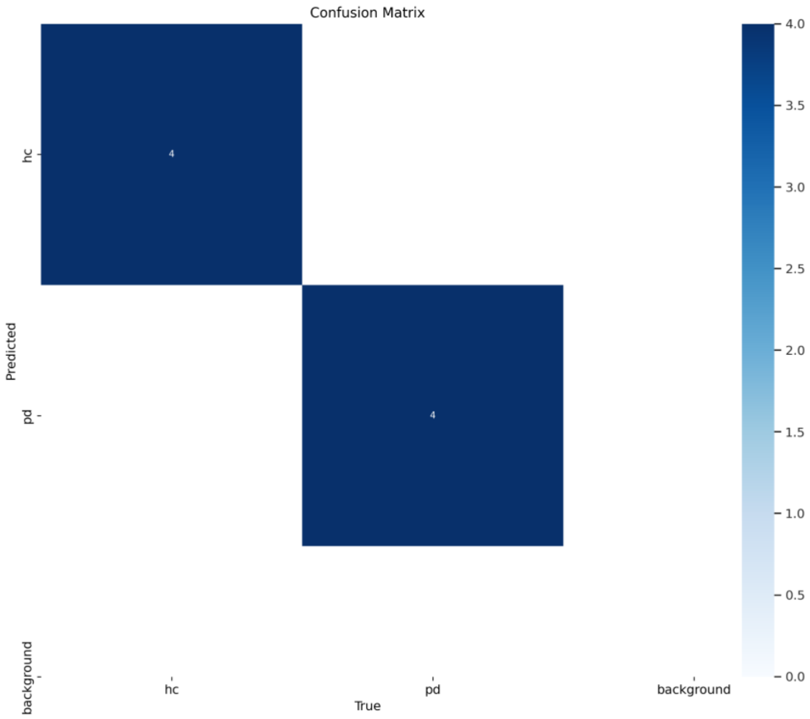

Figure S6. Confusion matrix of YOLO for test dataset for the 4th round of permutations.

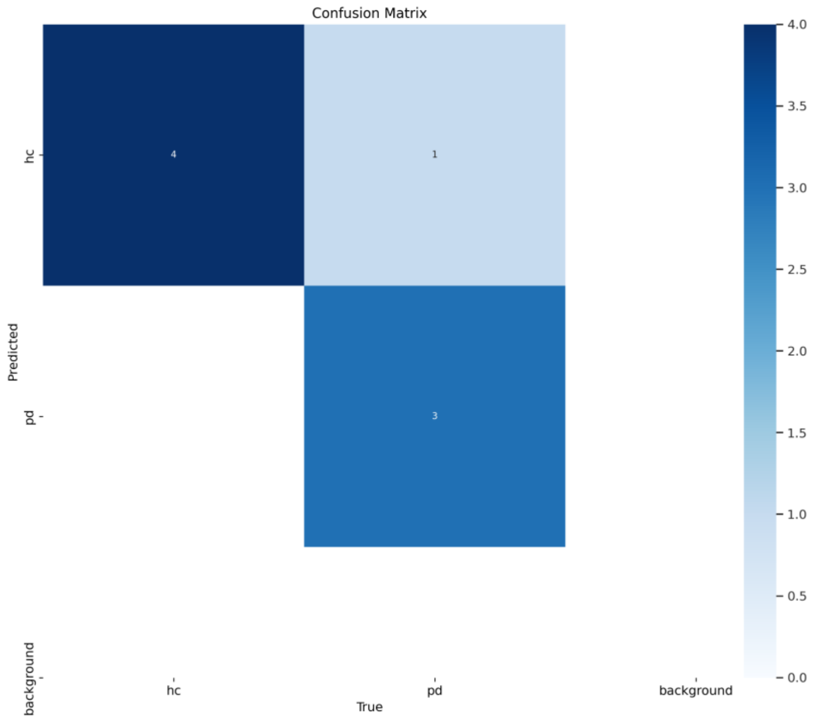

Figure S7. Confusion matrix of YOLO for test dataset for the 5th round of permutations.

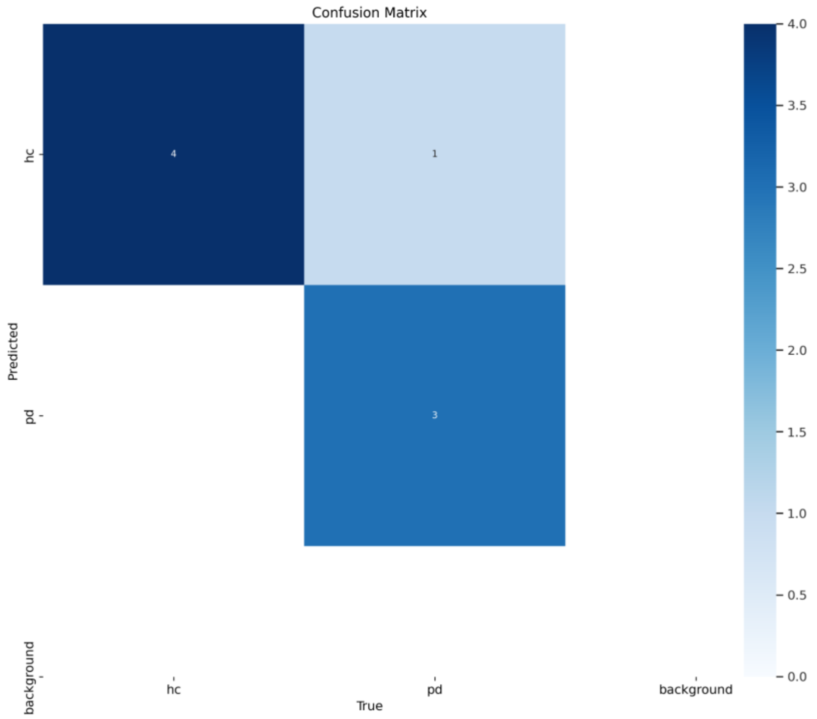

Figure S8. Confusion matrix of YOLO for test dataset for the 6th round of permutations

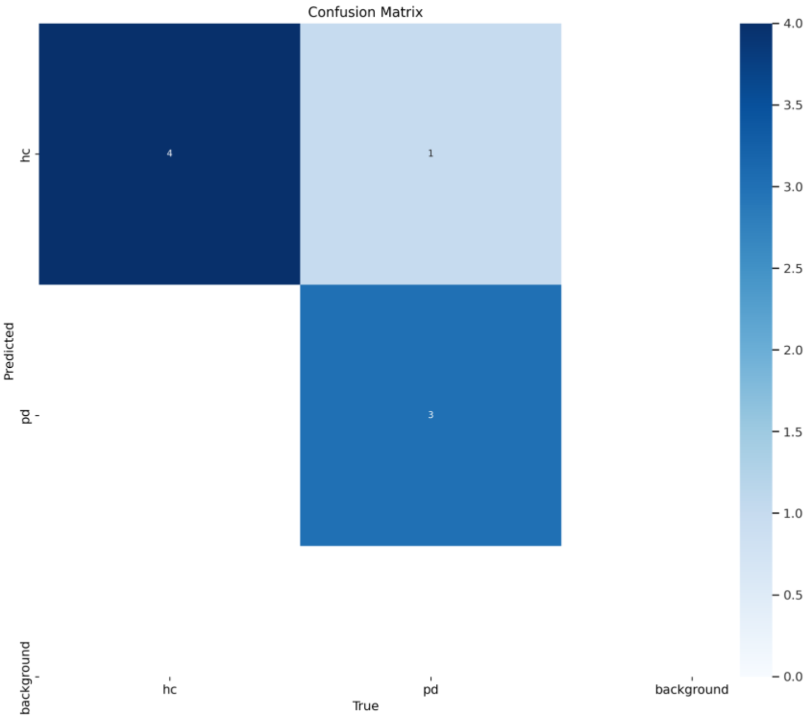

Figure S9. Confusion matrix of YOLO for test dataset for the 7th round of permutations

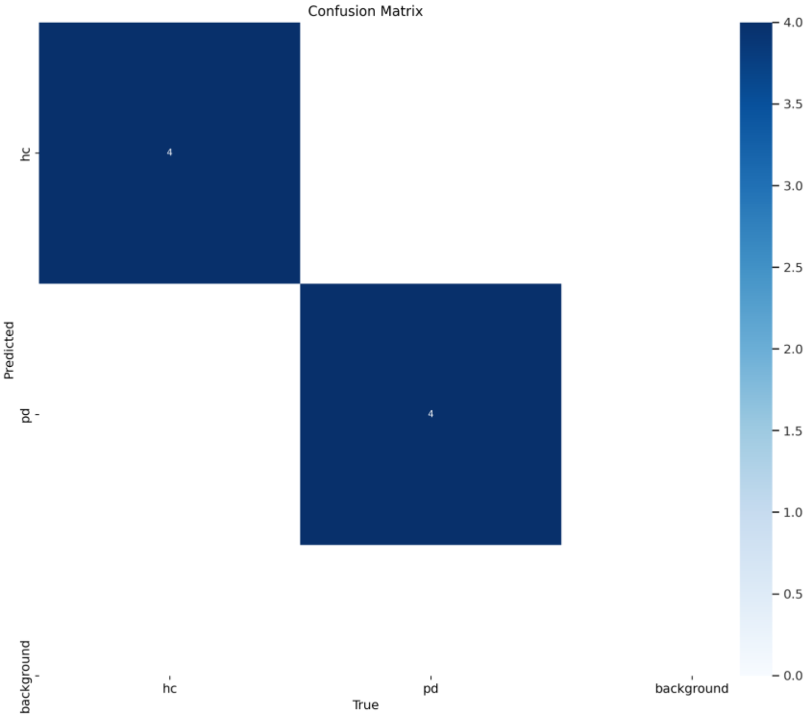

Figure S10. Confusion matrix of YOLO for test dataset for the 8th round of permutations

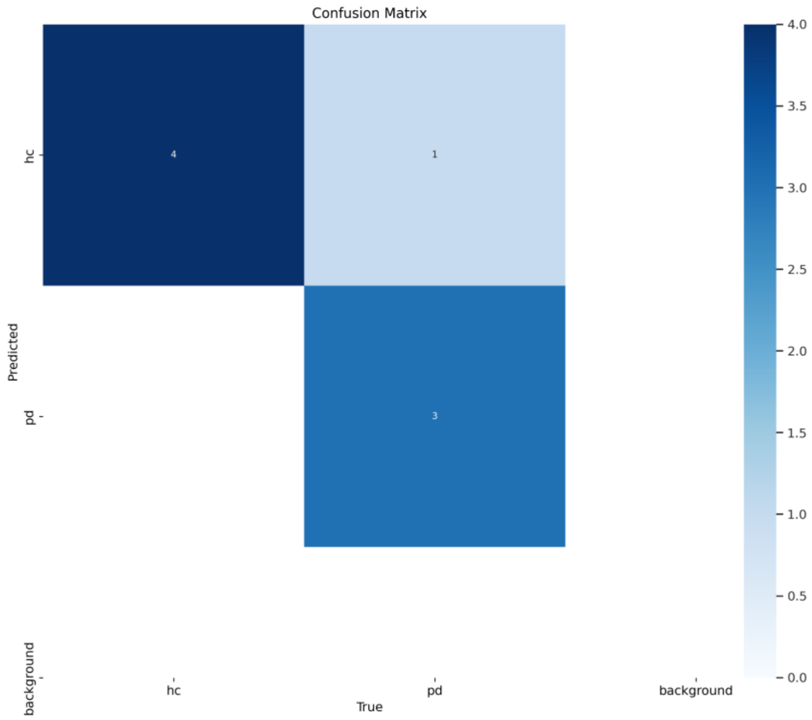

Figure S11. Confusion matrix of YOLO for test dataset for the 9th round of permutations

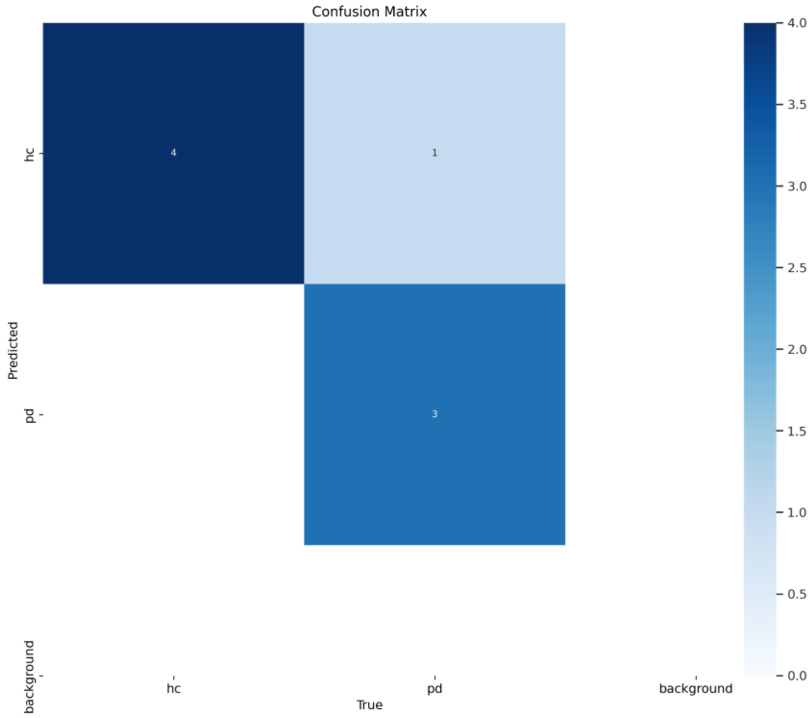

Figure S12. Confusion matrix of YOLO for test dataset for the 10th round of permutations
